# Commentary: Case report: Successful intravenous lipid emulsion therapy for canine amphetamine toxicosis

**DOI:** 10.3389/fvets.2022.1011210

**Published:** 2022-09-16

**Authors:** Ju-Tae Sohn

**Affiliations:** ^1^Department of Anesthesiology and Pain Medicine, Gyeongsang National University College of Medicine, Gyeongsang National University Hospital, Jinju-si, South Korea; ^2^Institute of Health Sciences, Gyeongsang National University, Jinju-si, South Korea

**Keywords:** lipid emulsion, toxicity, dosage, drug, local anesthetic, allometric scaling

## Introduction

I read with interest a case report titled “Case Report: Successful Intravenous Lipid Emulsion Therapy for Canine Amphetamine Toxicosis” recently published in Frontiers in Veterinary Science (1). Lipid emulsions have been reported to treat drug toxicity caused by non-local anesthetics in veterinary medicine, including lamotrigine, ivermectin, minoxidil, and naproxen (2–5). I would like to share my comments regarding the lipid emulsion dosage used for treatment for amphetamine toxicity in this case report involving dogs.

## Dosage for lipid emulsion resuscitation in human

Lipid emulsions are routinely used to treat systemic toxicity caused by local anesthetics in humans (6). The lipid emulsion dosing regimen used for systemic toxicity of local anesthetics in humans is as follows: bolus administration with a 1.5 mL/kg lipid emulsion (20%) followed by a 0.25 mL/kg/min lipid emulsion (20%) (6, 7).

## Discussion

As the authors stated, the recommended lipid emulsion dosage used for drug toxicity in veterinary medicine is unavailable. However, the conversion of human drug dose into animal equivalent dose should consider allometric scaling, which takes into account the difference in body surface related to body weight between humans and animals (8). The extrapolation of human drug dosage to animal equivalent dosage is performed based on the normalization of drug dosage on the body surface area (8). Generally, the metabolic rates and physiological processes of large animals are lower and slower than those of small animals, respectively (8). In addition, based on body weight, large animals require a lower drug dose than small animals (8). Thus, based only on body weight, dosage calculation from humans to animals is not a rational approach (8). However, authors described “An initial bolus of 1.5 ml/kg of IVLE was administered over 5 min, followed by a 0.25 ml/kg/min CRI over 1 h” in this case report, which is in agreement with lipid emulsion dosage used for the treatment of systemic toxicity caused by local anesthetics in human (1, 6, 7). As the metabolic rate increases with an increase in the body surface area, considering the body weight and body surface of each species, the ratio of the correction factor (K_m_: kg/m^2^), which is average body weight divided by body surface area, is used for drug dosage conversion between species (8). The K_m_ human (for example, weight: 60 kg, and body surface area: 1.62 m^2^) and dogs (for example, weight: 24 kg, body surface area: 0.84 m^2^) is estimated to be ~37 (60 divided by 1.62) and 28 (24 divided by 0.84), respectively (8–10). Thus, the K_m_ ratio used for drug dosage conversion from humans to dogs is estimated to be ~1.32 (K_m_ of human divided by K_m_ of dog; 37 divided by 28). The animal equivalent dose was calculated from the human dose using the following method: animal equivalent dose (mg/kg) = human dose (mg/kg) × K_m_ ratio (K_m_ of human divided by K_m_ of animal) (8, 10). Taking this method into consideration, the approximate dog (body weight: 24 kg) equivalent dose of lipid emulsion (20%) used for treatment of systemic toxicity induced by local anesthetic in humans is as follows: 1.98 mL/kg (1.5 mL/kg × 1.32) bolus administration followed by 0.33 mL/kg/min (0.25 mL/kg/min × 1.32) (7–10). In addition, previous laboratory experiments used bolus administration of 9 mL/kg lipid emulsion (20%) for lipid emulsion treatment in an animal study using rats, which is equivalent to bolus administration of lipid emulsion (1.5 mL/kg) used for the systemic toxicity of local anesthetics in humans (7, 11, 12). I surmised that this 9 mL/kg bolus administration of lipid emulsion (20%) was calculated using following method: lipid emulsion dosage used in the rat is human dosage (1.5 mL/kg) multiplied by K_m_ ratio (~6; K_m_ [37] of human divided by K_m_ [6] of rat) (8). Intravenous administration of local anesthetics causes systemic toxicity. However, in veterinary medicine, drug toxicity caused by non-local anesthetics is induced by oral administration. These two situations differ in terms of toxicokinetics. Thus, further studies are required to determine the optimal dosing regimen of lipid emulsions to treat drug toxicity in veterinary medicine.

## Author contributions

J-TS: conceptualization, writing—original draft, writing—review, and editing.

## Conflict of interest

The author declares that the research was conducted in the absence of any commercial or financial relationships that could be construed as a potential conflict of interest.

## Publisher's note

All claims expressed in this article are solely those of the authors and do not necessarily represent those of their affiliated organizations, or those of the publisher, the editors and the reviewers. Any product that may be evaluated in this article, or claim that may be made by its manufacturer, is not guaranteed or endorsed by the publisher.
